# Industrial Cooling Tower Disinfection Treatment to Prevent *Legionella* spp.

**DOI:** 10.3390/ijerph14101125

**Published:** 2017-09-26

**Authors:** Matteo Iervolino, Benedetta Mancini, Sandra Cristino

**Affiliations:** Department of Biological, Geological and Environmental Sciences, BiGeA, University of Bologna, via San Giacomo 12, 40126 Bologna, Italy; matteo.iervolino2@unibo.it (M.I.); benedetta.mancini2@unibo.it (B.M.)

**Keywords:** cooling tower system, disinfection treatments, microbiological parameters, *Legionella* spp.

## Abstract

The contamination of industrial cooling towers has been identified as one cause of legionellosis, but the real risk has been underestimated. Two different disinfection treatments were tested on *Legionella* colonization in an industrial Cooling Tower System (CTS). Environmental monitoring of *Legionella*, *P. aeruginosa*, and a heterotrophic plate count (HPC) at 36 °C was performed from June to October 2016. The disinfection procedures adopted were based on hydrogen peroxide (H_2_O_2_) and silver salts (Ag^+^), in addition to an anti-algal treatment, then using hyperclorination as a shock, and then continuous treatment by sodium hypochlorite (NaClO). *L*. *pneumophila* serogroup 8 was found at a concentration of 5.06 Log cfu/L after the CTS filling; a shock treatment performed by H_2_O_2_/Ag^+^ produced a rapid increase in contamination up to 6.14 Log cfu/L. The CTS activity was stopped and two subsequent shock treatments were performed using NaClO, followed by continuous hyperclorination. These procedures showed a significant decrease (*p* < 0.05) in *Legionella* concentration (1.77 Log cfu/L). The same trend was observed for *P*. *aeruginosa* (0.55 Log cfu/100 mL) and HPC (1.95 Log cfu/mL) at 36 °C. Environmental monitoring and the adoption of maintenance procedures, including anti-scale treatment, and physical, chemical, and microbiological control, ensure the good performance of a CTS, reducing the *Legionella* risk for public health.

## 1. Introduction

Cooling towers are used in most large commercial and residential buildings, industrial power generation units, and in chemical, petrochemical, and petroleum industries to disseminate waste heat into the environment. These water systems provide an environment highly favorable to microbial growth [1,2] and have been determined to be a source for dissemination of human pathogens, especially *Legionella* spp., as well as other pathogenic bacteria, protozoa, and viruses [3,4]. *Legionella* show a ubiquitous distribution in aqueous environments and occur often in technical water carrying systems. The microorganism grows normally in natural aquatic matrices or in various artificial systems in a temperature range between 25 °C and 42 °C.

The genus *Legionella* is a set of Gram negative bacteria which includes approximately 61 species with at least 79 serogroups. The most dangerous species, in 90% of cases that causes legionellosis, is the *L*. *pneumophila* serogroup 1 [5,6,7,8].

Despite mandatory reporting for legionellosis in several countries, the true number of cases is underestimated. In Italy, the incidence of disease in 2015 was equal to 25.8 cases per million. The data show that out of 1569 cases reported, 82 (5.3%) had been admitted to hospital, 200 cases (12.7%) were travel-associated, 38 cases (2.4%) were living in day care centers, and 13 cases (0.8%) had other risk factors [9]. Twenty cases were community-acquired cases of pneumonia (1.3%) from swimming pool visitors and 16 (1.0%) cases were due to dental care treatments. The remaining 76.5% of cases were not associated with a specific risk factor, but the ubiquity of *Legionella* in natural and artificial environments permit their classification as community acquired cases.

In artificial water systems, the presence of stagnation, biofilm, and scale enhances *Legionella* survival and facilitate its multiplication [10]. The inhalation of aerosolized particles is a unique form of acquiring the microorganism, thanks to its ability to colonize the lower respiratory tract and start infection in the alveolar macrophages [11]. Therefore, a correlation between the number of colony forming units (cfu) in water or in air, and the risk of infection cannot be specified [12].

The literature and official reports have well-documented the environmental conditions contributing to the transmission and aspiration of *Legionella*; artificial water systems, such as supply facilities, heated reservoirs, and cooling towers, represent potential sources of human infection [8,10,13,14,15].

Cooling tower systems (CTSs) represent an important source and reservoir for *Legionella* growth; they have a large volume of water open to the atmosphere, a temperature maintained between 20 °C and 35 °C, and they contain some elements such as carbon and nutrients from atmospheric sources. CTSs also have a building water loop, and have non-chemical treatments that are used in cooling towers, including anti-scale, corrosion inhibitors, and surface-attached microbial communities (biofilms) that can support microbial growth [10,16,17,18].

The aerosolized water that comes from cooling towers that enters urban towns is often contaminated by microorganisms including *Legionella*, such as *Pseudomonas aeruginosa*. In the approximately 13% *Legionella*-positive samples, 36% were *Pseudomonas*-positive samples. However, aerosolized particles containing *Legionella* are not released continuously, and a lack of CTS maintenance procedures can be associated with an increased release of *Legionella* into the environment [10].

The mechanism that explains the *Legionella* release from biofilm via aerosol, leading to distribution into the environment, is mostly unknown. Different studies have well-documented the *Legionella* transport and the distances carried, depending on meteorological conditions, such as thermal inversion and the season. These problems are strictly associated with CTSs, which are widely distributed and have been consistently linked to community-acquired legionellosis outbreaks [13,19,20].

The CTS cleaning procedures play an important role in the prevention and spread of *Legionella*. During opening procedures after emptying the system, all of the interior components must be inspected to determine the type of treatment or the procedures required. All of the debris and sludge is removed from the collection tank. The exchange pack should be cleaned or replaced if it is dirty or clogged. The water distribution system and drop separators should be thoroughly cleaned and inspected to check for any damage or missing parts. Even silencers or other accessories should be cleaned if there are signs of dirt. The CTS should be washed after cleaning and filled with clean water. An appropriate amount of chemical should be added, including biocide reagents, before re-starting the machine [7,21].

National and local guidelines recommend performing a disinfection procedure before opening the CTS after a prolonged closing, after each routine cleaning, or when significant changes to the operation system have occurred. The disinfection treatment of the CTS is necessary, especially when bacteriological contamination levels are high or as a preventive procedure to avoid biofilm formation [22,23,24].

Disinfection must be carried out according to appropriate procedures while ensuring the safety of the operators. Many physical, thermal, and chemical disinfection methods that either kill bacteria, producing a bactericidal effect, or inhibit the growth of bacteria without killing them, with a bacteriostatic effect, have been proposed. Although the advantages and disadvantages of these methods have been reviewed, no definitive recommendations have been provided [13,25].

The efficiency of disinfection treatments and maintenance procedures are important to control the growth of microorganisms, such as *Legionella* and *P*. *aeruginosa*, and Heterotrophic Plate Count (HPC) in CTS environments. Typical operating conditions maintained in a recirculating cooling system, including elevated temperature, near neutral pH, continuous aeration, and sunlight, provide ideal conditions for biological growth [26]. The 2007 WHO guidelines and different authors have recommended CTS disinfection procedures to adopt for *Legionella* control [13,27,28,29,30].

These methods address metal ions (copper and silver), UV light, and oxidizing and non-oxidizing agents. Oxidizing agents include chlorine, bromine, iodine, chlorine dioxide, chloramines and halogenated hydantoins, ozone, and hydrogen peroxide. Non-oxidizing agents include heterocyclic ketones, guanidines, halogenated amides and glycols, thiocarbamates, amines, aldehydes, thiocyanates, organotin compounds, and other agents [25,31,32]. Oxidizing antimicrobials are often effective when fed continuously using metering systems with small pumps, and many towers are successfully treated with shock or continuous dosage following the recommendations of different guidelines [13].

Continuous and shock treatments are two measures recommended to control *Legionella* in CTSs. Continuous treatments maintain low concentrations of disinfectant over a long period, while shock treatments use high doses of the product for short periods. Chlorine is the best-known disinfectant. It has been widely used to disinfect water systems, cooling towers, and other water installations [25,33]. However, the lack of effectiveness of hyperchlorination has been shown for completely eliminating *Legionella* from installations. Several authors have reported that the recolonization of hyperchlorinated systems can take place within weeks or months [34]. There are a large variety of other products that are also used in CTS treatments; limited information about the dosage and duration of activity has been reported and their action against *Legionella* is still poorly understood [27].

In this study, the effectiveness of two different disinfection treatments on the containment of *Legionella* spp. was evaluated. The presence of *P*. *aeruginosa*, one of the main components of biofilm [35] and HPC at 36 °C, commonly used as an indicator of water quality [36], were also studied in order to understand the microbiological dynamic in CTS water that could support *Legionella* growth. The physical and chemical parameters were also measured to determine the disinfectant dosage and to control CTS operating conditions. The analyses were performed during the environmental monitoring on a CTS, serving a metalworking company during its operating season (June–October 2016).

## 2. Materials and Methods

### 2.1. Cooling Tower Characteristics

In a metalworking industrial site located in Bologna, Italy, a CTS was installed in 2002 to serve an air conditioning circuit.

This CTS was reopened by the stakeholders in 2014, after five years of the building being closed. Starting in 2016, according to the company, the cooling tower was inserted into a risk assessment plan for *Legionella* containment and control, involving on a monthly monitoring during the whole period of its operation.

The CTS is an open cooling tower, series 3000 model S 3864, produced by Baltimore Aircoil Company, (BAC), (Jessup, MD, USA). The CTS has a water capacity of 12 m^3^, a power of 1755 KW (1,500,000 Kcal/h), with a water consumption of 3.5–3.7 m^3^/h, and a 2500 L/h evaporation value. The outdoor unit steel panels and structural elements consist of heavy-gauge galvanized iron steel. For the casing panels, polyester reinforced with UV-resistant fiberglass are used as a means of heat exchange. The patented exchange pack with integrated BACross^®^ eliminator drops has Eurovent certification. This exchange pack, containing individual sheets, efficiently eliminates water splashes allowing winter operation without frost, as shown in lab tests. These sheets are easily inspected and easy to clean inside of the tower as disassembly is not required. The ventilation system consists of two corrosion-resistant pulleys, a strap, and a motor. Along with the heavy-duty shaft fan bearings and the BAC Impervix engine, it ensures optimum operating efficiency throughout the year. Generally, one or more corrosion-resistant aluminum fans are fitted, contained in cylinders, and fitted with removable protection. The air inlet is plastic combined with UV-resistant input screens. The entrance of sunlight is blocked to prevent organic growth within the tower. The water distribution system consists of a low-pressure gravity pump with a water distribution tank with large, non-clog plastic nozzles to ensure uniform water distribution. The cleaning and washing of these nozzles and tanks is easy to perform. Bulkheads inside of the hot tub allow for a variable flow, thus saving 50% of the energy required for the pump and ensuring frost-free operation. An inclined cold-water tank has a hinged inspection door with an inward opening, anti-vortex, and re-intake filters, both easily accessible from the inside of the unit, and an optional internal walkway for easy access to the inside of the unit [37].

### 2.2. Cooling Tower Structure and Functional Operations

**Figure 1:**
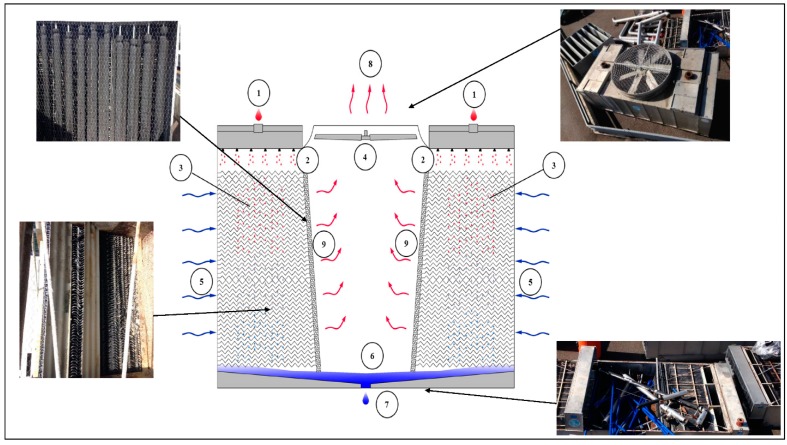
Cooling Towers System (CTS) characteristics, components, and principal operations.

As shown in Figure 1, warm process water (1) from the heat source enters into the water distribution system (2) at the top of the cooling tower on both sides where it is distributed over the fill or heat transfer media (3). At the same time, the axial fan located at the top of the unit (4), draws the air (5) from the sides of the unit over the fill. While the warm process water contacts the cold air, the latter heats up and part of the process water is evaporated which removes the heat from the remaining water. The sloping sump (6), or CTS basin, collects the cooled water after which it returns to the heat source of the process (7). The warm saturated air (8) first passes through the drift eliminators (9), which remove water droplets from the air, and then exits the tower at the top [37].

### 2.3. Water Sample Collection and Microbiological Analyses

Five liters of cold water were collected in sterile polytetrafluoroethylene (PTFE) bottles containing a sodium thiosulfate solution (10%, *v/v*) to neutralize any residual disinfectant. All water samples were analyzed for *Legionella* spp., *P*. *aeruginosa*, and HPC at 36 °C.

**Figure 2:**
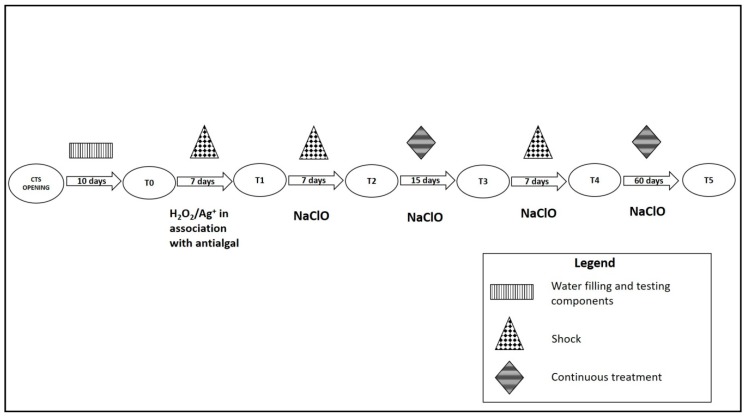
CTS disinfection treatments performed during the sampling period.

A total of six monitoring sessions were performed from June to October 2016. For each monitoring session, five samples, each one of 1 L, were collected from different points of CTS basin. The monitoring session corresponded to different disinfection treatments. T0 was 10 days after CTS opening procedures: filling with municipal water and testing of the electronic components. T1 was seven days after shock by hydrogen peroxide and silver salts (H_2_O_2_/Ag^+^), along with an anti-algal. T2 was seven days after the first shock by sodium hypochlorite (NaClO) and continuous treatment. T3 was 15 days after the first shock by NaClO and continuous treatment. T4 was seven days after the second shock by NaClO and changing the dosage of continuous treatment during the day. T5 was two months after the previous disinfection procedure maintenance (Figure 2).

Microbiological analyses for *Legionella* detection were performed by a cultural method in accordance with ISO11731-1998 [38]. Isolates were identified on the basis of culture, biochemical properties, and serological characteristics. The culture was determined with differential growth in buffered charcoal yeast agar, with and without L-cysteine (BCYE and BCYE-L-cys). The biochemical properties included fluorescence, oxidase activity, catalase activity, the ability to hydrolyze hippurate, and β-lactamase activity. The serological characteristics included using a *Legionella* latex test kit (ThermoFisher Scientific, Oxoid Ltd., Basingstoke, UK). Serogroups were also identified by agglutination tests using commercial antisera (Polyclonal latex reagents; Biolife, Milan, Italy). The results are expressed as a mean of Log cfu/L.

The samples were simultaneously analyzed for the presence of *P*. *aeruginosa* growth, which is a strong competitor of *Legionella* spp. [35] in water environment, contributing to biofilm formation. The analysis was carried out using the standard Membrane Filter Technique, according to UNI EN ISO 16266:2008 [39] using *Pseudomonas* selective agar (PSA) (Biolife, Milan, Italy).

Suspected colonies grown on the selective media were sub-cultured and identified using the Crystal Enteric/Non-Fermenter ID kit (Crystal E/NF) (Becton Dickinson, Cockeysville, MD, USA), according to the manufacturer’s instructions [40]. The results are expressed as a mean of Log cfu/100 mL.

Finally, an analysis was used to determine HPC at 36 °C, in order to control the total biological contamination in the CTS. The analysis was performed according to UNI EN ISO 6222:2001, using the standard plate method on Tryptic glucose yeast agar (PCA) (Biolife, Milan, Italy) [41]. The results are expressed as a mean of Log cfu/mL.

### 2.4. CTS Disinfection Procedures

Two different disinfection treatments were adopted: the first was performed for seven days (T1) based on H_2_O_2_/Ag^+^. A shock disinfection treatment was set up using a dosing pump to add a concentrated product at a concentration of 100 mg/L to the water in the CTS. An anti-algal treatment was added, based on quaternary ammonium salts, which are known for their anti-algal, anti-corrosive, and anti-fouling biocide functions, at a dosage of 0.005 mg/L.

The second treatment was performed after replacement of the H_2_O_2_/Ag^+^ and anti-algal treatment. A new device was installed to treat the CTS with NaClO. Two subsequent shock treatments (T2 and T4) were performed at a concentration of 50 mg/L for 2 h, in accordance with the Italian Guidelines for *Legionella* containment and control [22], along with an anti-scale and corrosion inhibitor treatment, a liquid product based on natural mineral salts such as orthophosphates, polyphosphates, and alkaline silicates dosed at 0.1 mL/L.

After the shock, the NaClO device was setting to perform a continuous treatment at a dosage of 2–3 mg/L. The continuous dosage adopted was changed in relation to the microbiological results obtained during the monitoring. Total and residual chlorine were monitoring during treatment by measurements carried out in the CTS.

### 2.5. Physical and Chemical Analyses

All physical and chemical parameters were recorded daily in the CTS basin, other than during the monitoring sessions. The values reported in Table 1 and Table 2 represent the mean values of all data recorded.

The temperature (°C) was measured using a conductivity meter coupled with a thermistor probe (Temp 5 basic for probe Pt100 RTD from −50 °C to +199 °C, Eutech Instruments Pte Ltd., Singapore). pH was measured with CWT AQUATEST.

The water conductivity (microsiemens µS/cm) was measured by a bleed-off system. This system consisted of a control unit, called PCR Multi Universal (Chillichemie, Bologna, Italy), that continuously determined the conductivity on the water drained from the CTS. The conductivity setting level determined the opening of the drainage valve and controlled the CTS water filling.

The hardness (°F) was measured daily in the CTS basin with IPT Total Hardness (Reasol, Srl, Milan, Italy) following the manufacturer’s instructions.

The hydrogen peroxide component of H_2_O_2_/Ag^+^ (mg/L) was measured on-site using a commercial kit. The kit uses a colorimetric test based on peroxidase activity to transfer peroxide oxygen to an organic redox indicator; this produces a blue oxidation product. The hydrogen peroxide concentration is measured semi-quantitatively by visual comparison of the result seen on the reaction zone of the test strip with the fields on a color scale, with a range of 0.1–200 mg/L H_2_O_2_.

Total and free chlorine (mg/L) were measured by a colorimeter (Orbeco-Hellige, Inc., 6456 Parkland Drive, Sarasota, FL, USA, Mini Analyst, Series 942, Model 942-001) from the CTS basin.

Results of the physical and chemical parameters measured during all testing periods are expressed as the mean value ± standard deviation (SD).

### 2.6. Statistical Analysis

Bacteriological data were converted into Log (x + 1) values to normalize non-normal distributions. The results were then analyzed using Student’s *t*-test (Stata 10 Data Analysis and Statistical Software; StataCorp LP, College Station, TX, USA). *p* values (*p*) < 0.05 were considered significant.

## 3. Results

### 3.1. Microbiological Analyses

**Figure 3:**
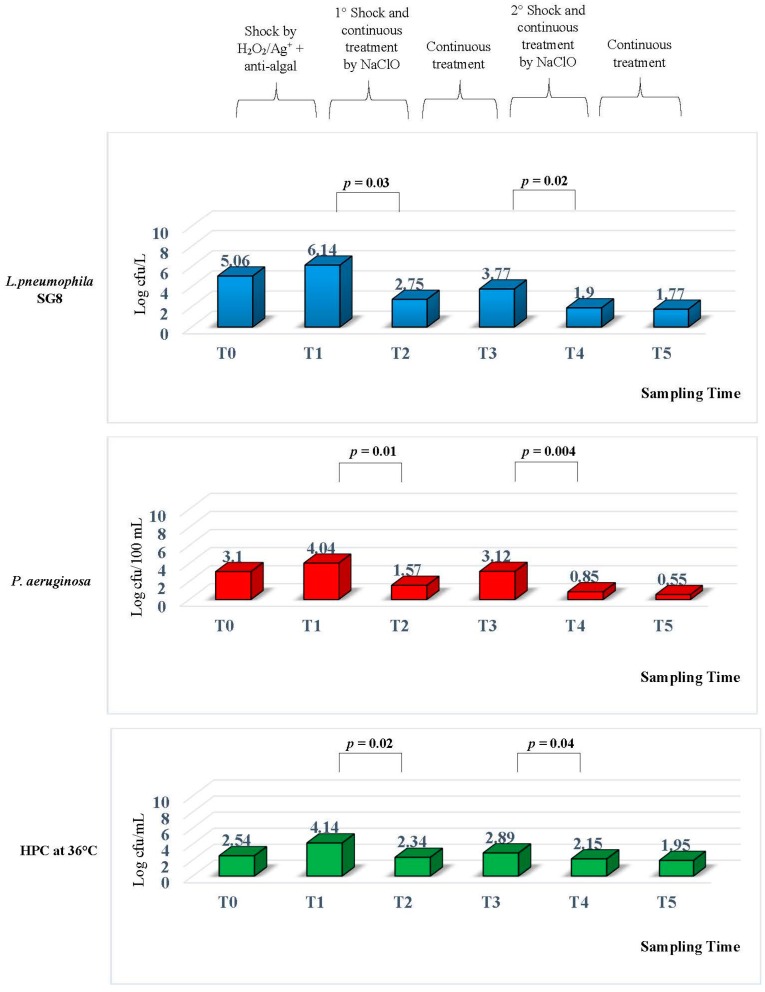
Mean values for the contamination by *L*. *pneumophila* SG8, *P*. *aeruginosa*, and heterotrophic plate count (HPC) at 36 °C in a CTS.

The levels of *Legionella*, *P*. *aeruginosa*, and HPC at 36 °C in the CTS were monitored during its operation at six different time points: T0, T1, T2, T3, T4, and T5. The microbiological parameters are shown in Figure 3.

When the CTS was opened at T0, when no treatment had been added, and the water in the CTS was a potable water was obtained from a Bologna aqueduct, we found a high level of microbiological contamination. *L*. *pneumophila* serogroups 8 (SG8) was at a concentration of 5.06 ± 0.042 Log cfu/L, *P*. *aeruginosa* had a concentration of 3.10 ± 0.28 Log cfu/100 mL, and HPC had a concentration level of 2.54 ± 0.28 Log cfu/mL at 36 °C.

These results suggested the introduction of a disinfection measure based on H_2_O_2_/Ag^+^ along with an anti-algal treatment. The control performed to test the efficiency of treatment T1 showed an increase of *L*. *pneumophila* SG8 contamination, reaching a concentration of 6.14 ± 0.14 Log cfu/L. The same data were found for the *P*. *aeruginosa* concentration, from 3.10 ± 0.28 Log to 4.04 ± 0.028 Log cfu/100 mL, and HPC levels rose from 2.54 ± 0.28 Log to 4.14 ± 0.14 Log cfu/mL at 36 °C.

The results obtained from T1 suggested that a replacement of the H_2_O_2_/Ag^+^ device would be required as well as the introduction of a second disinfection treatment based on NaClO at a shock concentration of 50 mg/L for two hours. After the shock treatment, the CTS circuit was washed and continuously treated with NaClO at a concentration of 2–3 mg/L.

At T2, seven days following the introduction of a new NaClO device, we found a decrease in all the microbiological levels. The statistical analysis performed between T2 and T1 showed a significant decrease for all parameters tested.

In particular, *L*. *pneumophila* SG8 had a concentration of 2.75 ± 0.21 Log cfu/L (∆Log 3.39) with *p* = 0.03 and *P*. *aeruginosa* showed a concentration of 1.57 ± 0.28 Log cfu/100 mL (∆Log 2.47) with *p* = 0.01. For the HPC contamination, the concentration had decreased to 2.34 ± 0.28 Log cfu/mL (∆Log 1.8) with *p* = 0.02.

After 15 days of this treatment, we performed the next monitoring T3 and we found a decrease of residual chlorine levels associated to an increase in *L*. *pneumophila* SG8 contamination, but the values found were lower than those observed at T0.

In fact, at T3, the *L*. *pneumophila* SG8 concentration was 3.77 ± 0.24 Log cfu/L, *P*. *aeruginosa* had a concentration of 3.12 ± 0.14 Log cfu/100 mL, and HPC had a concentration of 2.89 ± 0.14 Log cfu/mL at 36 °C.

In light of these results, we decided to perform a new shock based on NaClO at a concentration of 50 mg/L, through the same procedure previously described, but we decided to change the dosage during continuous treatment. The daily treatment, from 7:00 a.m. until 5:00 p.m. the level of NaClO was changed to 3 mg/L, and during the evening, from 6:00 p.m. until 6:00 a.m., where the concentration was set at 1.5 mg/L.

The monitoring performed at T4, seven days after the second NaClO shock, showed a reduction in all the tested microbiological parameters: *L*. *pneumophila* SG8 had a concentration of 1.9 ± 0.28 Log cfu/L, *P*. *aeruginosa* was 0.85 ± 0.14 Log cfu/100 mL, and HPC at 36 °C was 2.15 ± 0.14 Log cfu/mL.

The statistical analysis performed between T4 and T3 showed a significant decrease for all parameters, with *p* = 0.02 for *L*. *pneumophila*, *p* = 0.004 for *P*. *aeruginosa*, and *p* = 0.04 for HPC at 36 °C.

The conditions measured in T4 were maintained, until the last sampling performed at T5, two months later. The results showed that the concentrations of microbiological parameters were in line with those previously found. In particular, *L*. *pneumophila* SG8 displayed a concentration of 1.77 ± 0.24 Log cfu/L, *P*. *aeruginosa* was 0.55 ± 0.14 Log cfu/100 mL, and HPC had a concentration of 1.95 ± 0.14 Log cfu/mL at 36 °C.

These data demonstrated that the NaClO treatment worked significantly better than H_2_O_2_/Ag^+^ for reducing the biological growth in a CTS environment.

### 3.2. Physical and Chemical Analyses

The physical and chemical conditions of the water in the CTS tested during the monitoring period are reported in Table 1 and Table 2, respectively. The data are expressed as mean concentrations ± SD. Introducing H_2_O_2_/Ag^+^, at a shock concentration of 100 mg/L, along with an anti-algal treatment, did not change the water temperature, pH, conductivity, or hardness. A deviation in the conductivity values was observed after the shock treatment was performed with NaClO at T3, which required a technical operation on the CTS control panel. The parameters measured are in line with Italian regulations on discarding waste water [42].

## 4. Discussion

Environmental investigations of Legionnaires’ disease outbreaks linked to the CTS have revealed poorly maintained systems, a lack of control measures, and failure of system equipment. Regulations and many investigators have recommended the regular maintenance of the CTS based on the control of fouling, due to scale, salts, microbial growth, and the maintenance of its mechanical components. In addition, regular inspection and application of corrective measures to eliminate the parts of the system that facilitate water stagnation have been recommended. Further measures include the cleaning of all internal parts, such as the heat exchanger, and removing dirt, dust, dissolved solids, and organic materials. As described by Mouchtouri et al. [29], a great number of the CTS did not have a risk assessment and management plan in place, an operational manual available, or standardized cleaning and maintenance procedures, indicating that the CTS operation was not being systematically monitored.

This study is one of the first conducted in the Emilia Romagna region, before the *Legionella* epidemic event associated with a CTS in Parma occurred in October 2016 [43].

In Emilia Romagna, despite the 2008 regional guidelines and the emphasis placed on *Legionella*'s environmental and clinical surveillance, CTSs are an underestimated source of infections. As there is no register of the presence and location of these CTSs, the environmental control and the risk assessment plan is solely entrusted to the site itself. The reopening of the industrial site and the elaboration of a risk assessment plan for *Legionella* surveillance, has allowed us to monitor the CTS activities starting from the filling until closing operations (June–October 2016). The first sampling T0 showed the *Legionella* contamination level did not permit the start of CTS activity. The level of *L*. *pneumophila* SG8 found was higher than the limit prescribed by the *Legionella* national regulation, so we suggested a shock treatment that was performed with 100 mg/L of H_2_O_2_/Ag^+^ for seven days, with a mean peroxide residue in the CTS basin of 65 mg/L. To enhance the activity of the disinfectant, we also introduced an anti-algal compound, based on quaternary ammonium salts, for its anti-algal and anti-corrosive functions. The sampling performed seven days later, T1, showed that the treatment was inadequate for *Legionella* control, causing a rapid increase of one logarithmic unit. According to other studies and following our experience, often the addition of peroxide affects the movement of a biofilm, thus resulting in an increase in bacteria release. The presence of suspended (planktonic) biological growth increased the level of the potential aerosol emission, which may pose environmental and public health risks [44].

Hydrogen peroxide has several advantages over chlorine as an oxidizing biocide: it is compatible with different pipeline materials, and it does not react with the organic constituents in the water to form chlorine. However, at a continuous feed rate of approximately 2.5 mg/L, it was unable to control the planktonic population [45], and the silver can be deposited on the piping system, promoting a bacteriostatic effect. As described by different authors, H_2_O_2_ decomposes rapidly in different environmental conditions due to microbial catalase and peroxidase, and other than abiotic action, the decomposition is promoted by heavy metal, oxidative, and reductive reactions. The H_2_O_2_/Ag^+^ formulation is stable in high temperatures and its disinfection power increases significantly as the water temperature increases. The formulation has controlled *Legionella* well in hot water systems in a temperature range of 40–50 °C [44,46].

The water in CTS is cold, with a usual temperature of 28 °C. The circuit is open and the pipelines are made of galvanized iron steel that can support the decrease in H_2_O_2_/Ag^+^ oxidative power and an increase in the degradation reaction. These conditions could explain the decrease in H_2_O_2_/Ag^+^ effectiveness.

Given this data, the CTS activity was stopped and a visual inspection of various components was performed through the intervention of the manufacturer. During this inspection, we found some technical problems related to the CTS basin; it appeared heavily disintegrated with loose parts. Water stagnation and biofilm accumulation were easily seen. Some other problems were linked to the softener that showed a presence of *P*. *aeruginosa* colonies in the output, as well as drift eliminators packages that were dirty and weathered.

These problems could explain the high levels of HPC at 36 °C and *P*. *aeruginosa* detected at time T1. The parameters linked to an environment favourable to *Legionella* [36] showed a total decrease at T4, and the values were then in line with the national regulation limits (0 cfu/mL for HPC 36 °C and 0 cfu/100 mL for *P*. *aeruginosa*). The containment of these bacteria was maintained during the entire monitoring period, showing a good performance obtained due to the approach we used.

The increase in temperature (35–40 °C) in the summer leads to requests for the activation of the air conditioning system in the offices and in technical rooms makes it impossible to perform some CTS maintenance work in a short period. This suggested that replacing the disinfection system and a NaClO shock treatment, with a dosage of 20–50 mg/L, in the CTS basin were required. Before starting, a washing of the CTS circuit was performed by peracetic acid at a concentration of 20 mg/L as an anti-scale treatment, followed by the draining, flushing, and refilling of the CTS circuit with cold water. The CTS disinfection was subsequently performed by continuous NaClO treatment at a dosage of 2 to 3 mg/L. The sampling performed seven days after this treatment T2 showed a significant decrease in all of the microbiological parameters that were monitored.

After 15 days, to test the maintenance of the conditions previously described, we performed a new sampling T3, which displayed an approximate return to the initial contamination levels.

These data could be explained by the hot days alternating with the rainy days that occurred in Bologna during the testing period. The presence and release of *Legionella* is strictly associated to meteorological conditions. Different authors have shown that during hot summers, an increase in temperature may promote *Legionella* proliferation in fishponds, in the water of swimming pools, and cooling towers [47]. Especially in the CTS, *Legionella* can be aerosolized and transported over several kilometers within respirable vesicles, which are resistant to physical and chemical influences [10].

The chlorine measurements that were performed every two hours in the CTS basin and in the discharge water during sunny mornings showed a sudden decrease in the chlorine concentrations, due to the evaporation process occurring in the CTS open circuit. The analyses of data recorded by the conductivity control unit also displayed an increase of CTS conductivity values. The injection of solutes caused high values of conductivity, with an increase of CTS water drainage times. The discard of CTS water determined a decrease of the disinfectant concentration, reducing the contact time needed to control bacteria growth. To solve these problems, we decided to adopt two different chlorine dosages, through a specific automatic pump setting, in relation to environmental conditions. In particular, in the mornings when it was hot outside, the NaClO was injected at 3 mg/L from 7:00 a.m. to 6:00 p.m., while a dosage of 1.5 mg/L was injected from 6:00 p.m. to 8:00 a.m. This type of treatment was carried out for seven days until monitoring was performed at T4, and we found a reduction in *L*. *pneumophila* SG8 and the other bacteria tested. The monitoring of microbiological parameters was carried out after two months of treatment, and the last sampling performed at T5, two months after the introduction of NaClO disinfection, showed this action permitted the containment of all of the bacteria being monitored, especially *Legionella*. The amounts of bacteria remained in line with the prescribed regulatory limits. At this phase, it was important to train the staff involved in the control of the physical and chemical parameters in addition to the required maintenance procedures.

The physical and chemical parameters were monitored during all of the treatments to control the CTS operation conditions, and to ensure the limits established by Italian regulation for waste water discard were observed [42]. The results obtained did not show changes in CTS temperature conditions, pH, or hardness. In contrast, the presence of NaClO treatment led to an increase in the conductivity values that interfered with CTS water discard, as previously described. These considerations underline that the choice of disinfection treatment must align not only with microbiological factors, but must take into account the CTS operating conditions and environmental measures.

A specific register was created for the measurements performed two times per day directly by the company staff, to record the operation parameters of the CTS, including temperature, pH, conductivity, and hardness. The register allowed us to observe a good maintenance level in CTS activity until its closing.

## 5. Conclusions

CTSs are high efficiency air handling systems with lower energy consumption than refrigeration units, widely used in business activities. Where a CTS is present, especially if it has seasonal activity, the risk of bacterial contamination cannot be overlooked. Our data reveals that the preventive measures, including cleaning, disinfection treatments, chemical-physical, and microbiological analyses, must be undertaken before the opening of the system and during its entire operation. Rainwater stagnation, scale, and corrosion of the tower components can affect its functionality and increase the risk of the release of airborne microorganisms, such as *Legionella*. The adoption of a disinfection treatment is not always the best or only strategy for *Legionella* environmental surveillance; our data showed that the disinfectant can interfere with the CTS operating conditions. To prevent and control the CTS *Legionella* contamination, the best operations practices must be standardized, recorded, and often verified. Despite the encouraging results showed in this study, an important limitation is the small sample size. The ability to compare the same or different treatments with a large number of CTS samples could help to better determine the correct CTS disinfectant dosage to control the bacterial growth and to understand the mechanism that can support strain selection or resistance. The results in this study promote a preventive approach for the surveillance of *Legionella* in the other facilities with the CTS. The registration of the CTS, as prescribed in Italian regulation, is an important step that will help stakeholders adopt a risk assessment plan, supporting the environmental monitoring studies, and allow for the rapid identification of outbreak sources during epidemiological investigations.

## Figures and Tables

**Table 1:** Physical parameters tested of the CTS.

Time	Temperature (°C)	pH	Conductivity (µS/cm)	Hardness (°F)
T0	23 ± 1.41	9.15 ± 0.21	1550 ± 28.28	30 ± 5.66
T1	24.50 ± 0.71	8 ± 2.83	1700 ± 70.71	23 ± 2.83
T2	27.20 ± 0.28	6 ± 0.14	1610 ± 21.21	29 ± 7.07
T3	23.40 ± 0.85	6.50 ± 0.99	2840 ± 49.50	32 ± 0.71
T4	28 ± 2.83	8 ± 0.42	1900 ± 56.57	26 ± 4.24
T5	23.20 ± 1.13	8.20 ± 0.57	1500 ± 14.14	32 ± 8.49

**Table 2:** Chemical parameters tested and disinfection procedures for the CTS.

Time	H_2_O_2_/Ag^+^ with Anti-Algal (mg/L)	H_2_O_2_ Mean ± SD (mg/L)	NaClO (mg/L)	Residual Chlorine Mean ± SD (mg/L)	Total Chlorine Mean ± SD (mg/L)
T0				0.14 ± 0.20	0.55 ± 0.14
T1	Shock concentration 100 mg/L	65 ± 7.07		0.22 ± 0.31	0.95 ± 0.49
T2			50 mg/L (shock treatment)	30 ± 7.07	
			2–3 mg/L (continuous treatment)	1.45 ± 0.07	1.91 ± 0.04
T3				0.04 ± 0.028	0.2 ± 0.14
T4			50 mg/L (shock treatment)	30 ± 7.07	
			2–3 mg/L (continuous treatment)	2.38 ± 0.42	3.42 ± 0.25
T5			2–3 mg/L (continuous treatment)	1.35 ± 0.18	2.95 ± 0.38
